# Transcriptome analyses based on genetic screens for Pax3 myogenic targets in the mouse embryo

**DOI:** 10.1186/1471-2164-11-696

**Published:** 2010-12-08

**Authors:** Mounia Lagha, Takahiko Sato, Béatrice Regnault, Ana Cumano, Aimée Zuniga, Jonathan Licht, Frédéric Relaix, Margaret Buckingham

**Affiliations:** 1 Genopole, Institut Pasteur, 28 Rue du Dr Roux, 75015 Paris, France; 2 INSERM U668, Département d'Immunologie, Institut Pasteur, 25 Rue du Dr Roux, 75015 Paris, France; 3 DBM/Center for Biomedicine, Basel, Switzerland; 4 Northwestern University, Chicago, USA; 5 UMRS787, INSERM-UPMC-Paris VI, Institut de Myologie, Faculté de Médecine Pitié-Salpétrière, 75634 Paris, France; 6 CNRS URA 2578, Département de Biologie du Développement, Institut Pasteur, 25 Rue du Dr Roux, 75015 Paris, France

## Abstract

**Background:**

Pax3 is a key upstream regulator of the onset of myogenesis, controlling progenitor cell survival and behaviour as well as entry into the myogenic programme. It functions in the dermomyotome of the somite from which skeletal muscle derives and in progenitor cell populations that migrate from the somite such as those of the limbs. Few Pax3 target genes have been identified. Identifying genes that lie genetically downstream of *Pax3 *is therefore an important endeavour in elucidating the myogenic gene regulatory network.

**Results:**

We have undertaken a screen in the mouse embryo which employs a *Pax3^GFP ^*allele that permits isolation of Pax3 expressing cells by flow cytometry and a *Pax3^PAX3-FKHR ^*allele that encodes PAX3-FKHR in which the DNA binding domain of Pax3 is fused to the strong transcriptional activation domain of FKHR. This constitutes a gain of function allele that rescues the *Pax3 *mutant phenotype. Microarray comparisons were carried out between *Pax3^GFP/+ ^*and *Pax3^GFP/PAX3-FKHR ^*preparations from the hypaxial dermomyotome of somites at E9.5 and forelimb buds at E10.5. A further transcriptome comparison between Pax3-GFP positive and negative cells identified sequences specific to myogenic progenitors in the forelimb buds. Potential Pax3 targets, based on changes in transcript levels on the gain of function genetic background, were validated by analysis on loss or partial loss of function *Pax3 *mutant backgrounds. Sequences that are up- or down-regulated in the presence of PAX3-FKHR are classified as somite only, somite and limb or limb only. The latter should not contain sequences from Pax3 positive neural crest cells which do not invade the limbs. Verification by whole mount *in situ *hybridisation distinguishes myogenic markers. Presentation of potential Pax3 target genes focuses on signalling pathways and on transcriptional regulation.

**Conclusions:**

Pax3 orchestrates many of the signalling pathways implicated in the activation or repression of myogenesis by regulating effectors and also, notably, inhibitors of these pathways. Important transcriptional regulators of myogenesis are candidate Pax3 targets. Myogenic determination genes, such as *Myf5 *are controlled positively, whereas the effect of *Pax3 *on genes encoding inhibitors of myogenesis provides a potential brake on differentiation. In the progenitor cell population, *Pax7 *and also *Hdac5 *which is a potential repressor of *Foxc2*, are subject to positive control by *Pax3*.

## Background

During embryonic development, the Pax family of transcription factors play important roles in cell type specification and organogenesis [1]. In vertebrates, Pax3 is a key upstream regulator of skeletal myogenesis. This paired-box homeo-domain transcription factor is present in myogenic progenitor cells of the developing muscle masses and also in the multipotent cells of the somites from which all skeletal muscles in the trunk and limbs derive. Somites form as segments of paraxial mesoderm following a rostral/caudal gradient on either side of the embryonic axis. Initially Pax3 is expressed throughout the epithelial somite and then becomes restricted to the dorsal domain, the dermomyotome, which maintains an epithelial structure. The ventral somite gives rise to bone and cartilage of the vertebral column and ribs, whereas the Pax3 positive cells of the dermomyotome give rise to other mesodermal derivatives, including derm, smooth muscle and endothelial cells, as well as skeletal muscle. Experiments in the chick embryo [2-4] and in the mouse [5] have shown that different cell types derive from a single Pax3 positive cell. Myogenic progenitors delaminate from the edges of the dermomyotome to form the underlying skeletal muscle of the myotome. As development proceeds, the central domain of the dermomyotome where Pax7, the paralogue of Pax3, is also expressed, loses its epithelial structure and these Pax positive cells enter the underlying muscle masses where they constitute a progenitor cell population for all subsequent muscle growth. In the absence of both Pax3 and Pax7, these cells fail to enter the myogenic programme and many of them die [6]. The hypaxial domain of the dermomyotome, where Pax3, but not Pax7, is mainly expressed in the mouse, is an important source of myogenic progenitors. At the level of the limb buds, cells migrate from this domain to form the skeletal muscle masses of the limb. In the absence of Pax3, these cells fail to delaminate and migrate and subsequently undergo cell death [1]. Pax3 therefore controls migration of myogenic progenitor cells from the somite, entry into the myogenic programme and survival.

In order to understand how Pax3 functions in the multipotent cells of the dermomyotome and subsequently in myogenic progenitors, it is necessary to characterize Pax3 targets. During myogenesis *in vivo *very few targets have been identified. Notably, *c-Met *has been proposed as a direct Pax3 target [7]. This gene encodes a tyrosine kinase receptor that interacts with HGF, required for the delamination, and probably also the migration, and proliferation of myogenic progenitors [8]. Pax3 activation of the *c-Met *promoter, although not fully demonstrated *in vivo*, provides an explanation for the absence of progenitor cell migration and limb myogenesis in *Pax3 *mutants. This is also consistent with rescue of the ectopic migration seen in *Pax3*^*PAX3-FKHR/+ *^embryos, when c-Met is absent [9]. Entry of Pax3/7 positive progenitor cells into the myogenic programme depends on the myogenic determination factors, Myf5 and MyoD. Analysis of regulatory sequences in the 5' flanking region of *Myf5*, led to the characterization of an element at -57.5 kb from the gene that is responsible for transcription in the limb buds and older hypaxial somite. Activation of this element depends directly on Pax3 [10]. The *MyoD *gene is also regulated by a Pax3/7 binding site [11], although this regulation has not been explored in an embryonic context. Pax7 has a more limited expression pattern than Pax3 in the mouse somite, however they probably share many of the same targets, as indicated by the embryonic phenotype of a *Pax3^Pax7/Pax7 ^*mouse line in which Pax7 replaces Pax3 [12]. Further Pax3/7 targets have been identified using the C2 muscle cell line in which Pax3 or Pax7 was over-expressed [13,14]. In this context the *Myf5 *regulatory sequence targeted by Pax3 in the embryo was also shown to be a Pax7 target. *Id3*, which encodes a potential inhibitor of basic-helix-loop-helix transcription factors such as Myf5 or MyoD, was identified as a direct Pax3 target [14]. In the context of human Rhabdomyosarcomas, which result from a chromosomal translocation leading to the expression of a fusion protein, PAX3-FKHR or PAX7-FKHR in which the PAX DNA binding domain is followed by the strong transcriptional activation domain of the FOXO1A (FKHR) factor, a number of microarray screens have been performed on cultured cells (for review see [15]). Examples are provided by cDNA two colour arrays in which the authors identified genes differentially regulated by PAX3 or PAX3-FKHR over-expression in NIH3T3 cells [16], by Affymetrix arrays to find genes induced by PAX3 expression in a human medulloblastoma cell line [17], or by a casting approach of cyclic amplification and selection of cis-regulatory elements bound by human PAX3, PAX3-FKHR or murine Pax3 [18]. Very few target genes were common to these three approaches, probably reflecting the artificial conditions of the screens.

More recently, we have initiated a screen to systematically look for Pax3 targets in the mouse embryo. Since myogenic progenitors tend to die in the absence of Pax3, complicating the interpretation of a screen based on a comparison with material from *Pax3 *mutants, we used a gain of function approach. This was based on a *Pax3^PAX3-FKHR-IRESnlacZ/+ ^*(*Pax3^PAX3-FKHR/+^*) line that we had made, in which Pax3 targets such as *c-Met*, are over-activated. *Pax3^PAX3-FKHR ^*thus constitutes a *Pax3 *gain of function allele. We had previously shown that in *Pax3^PAX3-FKHR/Splotch ^*embryos (where *Splotch *is a spontaneously occurring *Pax3 *mutant allele) the *Pax3 *mutant phenotype is not observed, indicating that PAX3-FKHR can replace Pax3, which thus acts as a transcriptional activator in the myogenic context [9]. A *Pax3^GFP/+ ^*mouse line [6] permitted isolation of Pax3-GFP progenitor cells by flow cytometry, so that the transcriptomes of purified populations of *Pax3*^*GFP/*+ ^versus *Pax3*^*PAX3-FKHR/GFP *^cells could be compared by microarray analysis. This screen led to the identification of *Sprouty1 *and *Fgfr4 *shown to be a direct Pax3 target, and the demonstration that the self-renewal, versus entry into the myogenic programme, of myogenic progenitors is partly orchestrated by Pax3 modulation of FGF signalling [19]. *Dmrt2*, was also identified as a direct Pax3 target. This gene encodes a transcription factor, present in the Pax3 positive cells of the dermomytome, which regulates an early epaxial enhancer element of the *Myf5 *gene, required for the onset of myogenesis in the somite [20]. This screen also revealed that *Foxc2 *is negatively controlled by *Pax3*, and that this genetic repression is reciprocal in the epithelial somite and subsequently in the dermomyotome where these genes are co-expressed. Modulation of this equilibrium affects cell fate choices, resulting in Pax3 positive myogenic progenitors or Foxc2 positive vascular progenitors [21].

In this paper, we provide the first documentation of the *in vivo *gain of function screen for Pax3 targets and present data on other interesting candidates.

## Results and Discussion

### Experimental strategy and microarray results

**Figure 1:**
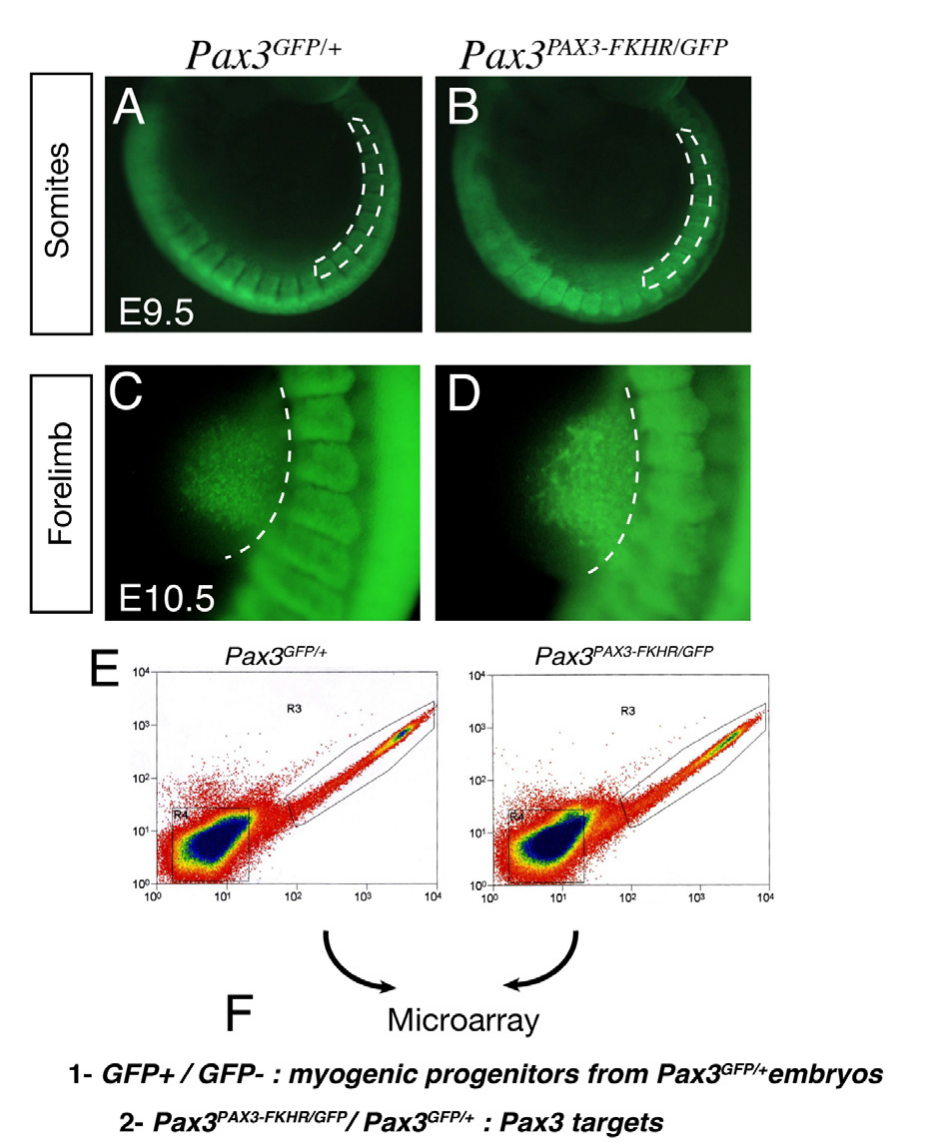
**Strategy of the screen for Pax3 targets**. (A-D) Embryos viewed under a fluorescence microscope from *Pax3^GFP/+ ^*(A, C) and *Pax3^PAX3-FKHR/GFP ^*(B, D) mouse lines at E9.5, focussing on the interlimb somites (A, C) and at E10.5 focussing on the forelimb bud (B, D). Dotted lines indicate the region dissected. (E) Isolation of GFP positive cells by flow cytometry from the two genotypes indicated, shown for material from E10.5 forelimb buds. The region R3, outlined in E, was used for transcriptome analysis of GFP positive cells. GFP negative cells were obtained from the R4 window shown in (E). (F) Microarrays were performed on RNA isolated from cells of interlimb somites and forelimb buds (A-D) with the genotypes indicated. The comparisons gave information about sequences that mark Pax3 positive myogenic progenitors (1) and that are candidate Pax3 targets (2).

Our strategy depended on the purification of Pax3 positive cells, which was implemented using a mouse line (*Pax3^GFP/+^*) in which one allele of *Pax3 *had been targeted with a GFP coding sequence [6], so that cells expressing this allele could be isolated by flow cytometry. This line was crossed onto the conditional *Pax3^PAX3-FKHR-IRESnlacZ/+ ^*(*Pax3^PAX3-FKHR/+^*) line [9] to produce *Pax3^PAX3-FKHR/GFP ^*embryos. After a further cross with a *PGK-Cre *transgenic line [22], this resulted in a *Pax3 *gain of function genetic background. In the crosses used here, *Pax3^PAX3-FKHR/GFP ^*embryos had a similar phenotype to *Pax3^PAX3-FKHR/+ ^*embryos. From our previous analysis of the *Pax3^PAX3-FKHR/+ ^*line [9], we know that PAX3-FKHR can save the *Pax3 *mutant phenotype, thus substituting for Pax3. This is accompanied by over-activation of known Pax3 targets such as *c-Met*, leading to some myogenic abnormalities. We used this allele to generate a gain of function genetic background. Somites in the interlimb region of E9.5 *Pax3^GFP/+ ^*(Figure 1A) and *Pax3^PAX3-FKHR/GFP ^*(Figure 1B) embryos were dissected to obtain the dorsal epithelial structure of the dermomyotome, which was cut away from the epaxial domain adjacent to the neural tube. This corresponds to a stage when most myogenic progenitors are still present in the dermomyotome, with cells delaminating from the epaxial dermomyotome to form the early skeletal muscle of the myotome. Care was taken to avoid Pax3 positive cells in the dorsal neural tube, although the presence of migrating neural crest cells from this source, that also express Pax3 [1], could not be excluded. Forelimb buds were dissected from *Pax3^GFP/+^*(Figure 1C) and *Pax3^PAX3-FKHR/GFP ^*(Figure 1D) embryos at E10.5, when Pax3 positive cells had migrated from the hypaxial domain of adjacent somites, but had not yet formed differentiated skeletal muscle in the forelimb buds. In this case, neural crest should be absent, since these cells do not enter the limb buds. After dissection, cells were dissociated from pooled samples of somites or forelimb buds from *Pax3^GFP/+ ^*and *Pax3*^*PAX3-FKHR/GFP *^embryos and separated by flow cytometry to obtain GFP positive fractions for microarray analysis (Figure 1E). In addition to the comparison of GFP+ cells from *Pax3^PAX3-FKHR/GFP^*; *Pax3^GFP/+^*embryos, GFP+/GFP- populations were compared from *Pax3^GFP/+ ^*embryos to identify sequences specific to Pax3 positive myogenic progenitors (Figure 1F). Obtaining enough material is a challenge at these embryonic stages, particularly from the forelimb bud which contains about 1000 Pax3 positive cells at E10.5, so that it was necessary to prepare material from >100 embryos with each genetic background in order to have enough material for cDNA synthesis, sample verification (see Additional file 1 Figure S1) and triplicate Affymetrix chip analyses.

Raw data were pre-processed to obtain expression values using the RMA (Robust Multichip Analysis) algorithm. Unreliable probe-sets called "absent" by Affymetrix Gene Chip Operating Software (GCOS) software (http://www.affymetrix.com/support/downloads/manuals/data_analysis_fundamentals_manual.pdf website) for at least 2 GeneChips out of 3 were discarded. LPE (Local Pooled Error) tests [23] were performed to identify significant differences in gene expression among *Pax3^PAX3-FKHR/GFP^*; *Pax3*^*GFP/+ *^and GFP+; GFP- samples. Benjamini-Hochberg (BH) [24] multiple-test correction was applied to control for the number of false positives with an adjusted 5% statistical significance threshold.

These data are available on the http://www.ncbi.nlm.nih.gov/geo/query/acc.cgi?acc=GSE22041 website.

Comparisons for E9.5 dermomyotome and E10.5 forelimb bud preparations are presented in Additional file 2 Tables S1-S3. Genes that are up-regulated (A) or down-regulated (B) in both somites and limb buds are shown in Additional file 2 Table S1. Additional file 2 Table S2 shows genes up-regulated (A) or down-regulated (B) for forelimb buds only, whereas Additional file 2 Table S3A and B shows such genes in somites only. Transcripts absent from the Pax3-GFP positive population, but observed in the presence of PAX3-FKHR are not included, since they may be due to non-PAX3 dependent FKHR activity. Transcripts that are present in Pax3-GFP positive cells and not detectable in the presence of PAX3-FKHR were retained. *Pax3 *transcripts are in this category and indeed provide a control, since the mouse gene is not transcribed in *Pax3*^*PAX3-FKHR/GFP *^embryos. The additional microarray screen, in which the transcriptome of the GFP negative cell population was compared to that of the Pax3-GFP positive population, gives an overview of transcripts that characterise myogenic progenitor cells of the forelimb bud, as shown in Additional file 3 Table S4.

Neural crest markers, such as AP2 gamma (Tcfap2c) or Ascl1 (also named Mash1) are present in the E9.5 dermomyotome lists and are also seen to a minor extent in the E10.5 limb samples, probably indicating the presence of some somitic material. This is also suggested by the presence of markers of differentiating muscle, such as skeletal muscle myosin or troponin, expressed at this stage in the myotome of the somite. The presence of markers of the sclerotome, such as Pax1 [25], probably reflects the inclusion of cells from the ventral somite compartment, perhaps due to some perduration of GFP, since the *Pax3^GFP ^*allele is expressed throughout the epithelial somite [21]. A gene encoding another typical marker of the sclerotome compartment, Uncx4.1 [26], was present in the list of Pax3 targets (Additional file 2 Table S1A), and also, unexpectedly, in the list of GFP+ specific genes for the limb bud (Additional file 3 Table S4). This may suggest that it is also expressed in myogenic progenitors, and indeed, the expression of *Uncx4.1 *is compromised in the absence of Pax3 (data not shown).

### Experimental validation of sequences of potential Pax3 targets modulated by PAX3-FKHR

**Figure 2:**
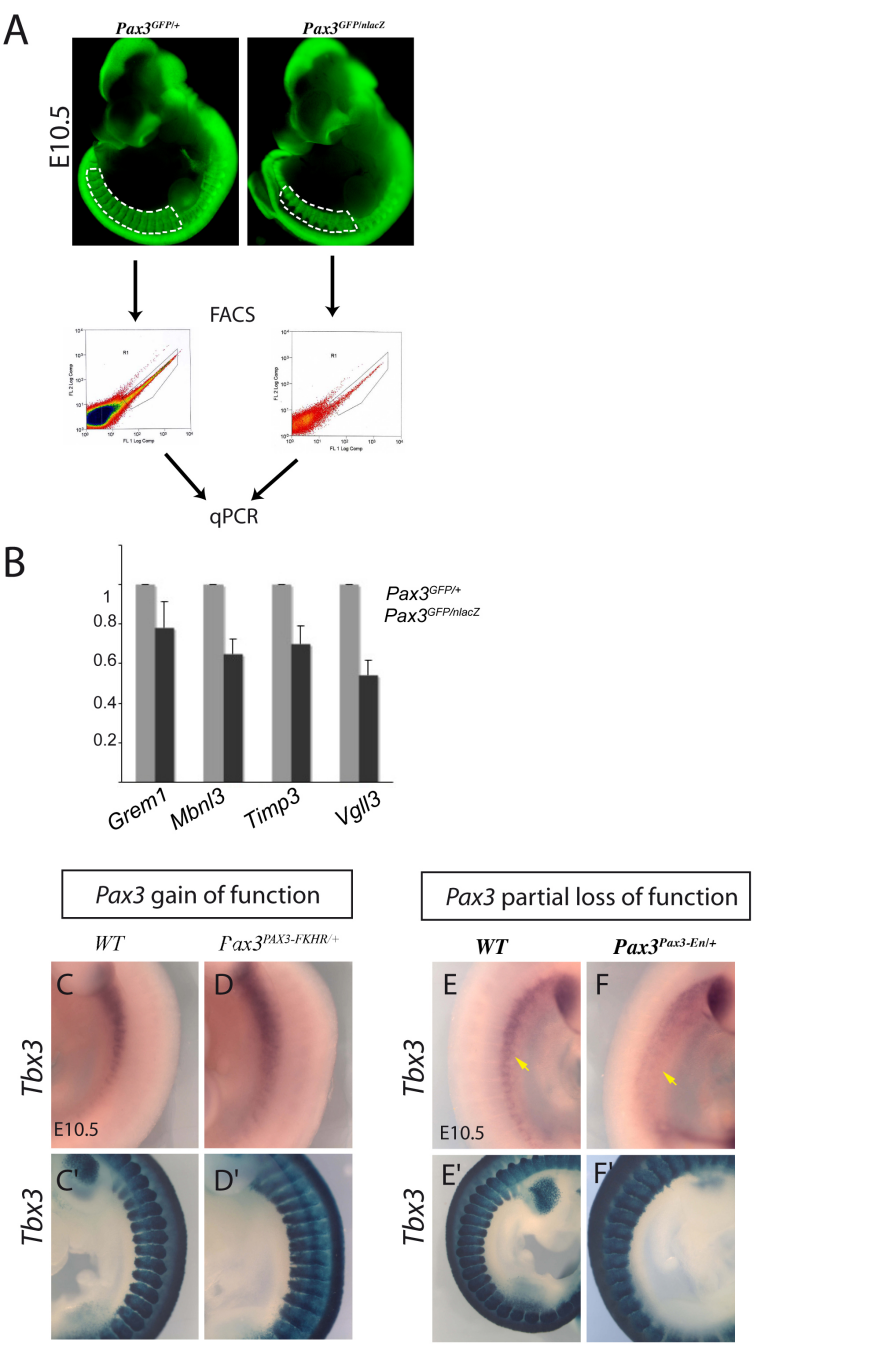
**Examples of validation on *Pax3 *loss of function genetic backgrounds**. Genes that emerged from the microarray analyses as potential Pax3 targets from the gain of function screen were checked on *Pax3 *loss of function genetic backgrounds. (A-B) Quantitative PCR analysis of transcripts in Pax3-GFP cells separated by flow cytometry (FACS) from interlimb somites of *Pax3^GFP/+ ^*and *Pax3^GFP/nlacZ ^*embryos at E10.5 (A). The same number of cells were analysed for each genotype and the results for *Gremlin1*, *Mbnl3*, *Timp3 *and *Vg113 *transcripts are presented as histograms relative to the *Pax3^GFP/+ ^*sample taken as 1 (B). In accordance with the microarray data, these genes are positively regulated by *Pax3*. (C-F) Whole mount *in situ *hybridization with a *Tbx3 *probe on control (C, E), *Pax3^PAX3-FKHR/+ ^*gain of function (D) and *Pax3^Pax3-En/+ ^*partial loss of function (F) embryos at E10.5, showing somites in the interlimb region. *Tbx3 *transcripts are high in the hypaxial somite domain, notably in more anterior somites, and their level depends positively on Pax3, as indicated by the microarray data. In the forelimb buds, there is extensive expression of *Tbx3 *in posterior mesenchyme which masks transcripts in Pax3 positive myogenic progenitors. In the lower panels, X-gal staining of β-galactosidase from *nlacZ *(C', E') (*Pax3^nlacZ/+^*) or *IRES-nlacZ *(*Pax3^PAX3-FKHR-IresnlacZ/+ ^*in D', *Pax3^Pax3-En-IresnlacZ/+ ^*in F') reporters shows the extent of the somites, notably the hypaxial domain which undergoes cell death in *Pax3 *mutants (F').

Genes that showed differences in expression on the *PAX3-FKHR *gain of function genetic background were validated by qRT-PCR on a *Pax3 *loss of function background. In order to avoid the problem of loss of cells due to cell death in the mutant, the same number of Pax3-GFP positive cells from *Pax3^GFP/+ ^*and *Pax3^GFP/nlacZ ^*embryos were analysed, after purification by flow cytometry (Figure 2A). Examples are shown in Figure 2B, for somites at E10.5. This is an important control to confirm that the increase in transcripts seen in the presence of PAX3-FKHR is not due to an effect of the fusion protein, other than that of strong transcriptional activation via Pax3 binding sites. Another control is provided by whole mount *in situ *hybridisation on different genetic backgrounds, which also demonstrates the localisation of cells that express the gene. In this case, the partial loss of function *Pax3^Pax3-En-IRESnlacZ/+ ^*(*Pax3^Pax3-En/+^*) line [10] was used, which expresses a fusion protein, with the Pax3 DNA binding domains fused to the repression domain of Engrailed. This results in down-regulation of Pax3 targets with the advantage that there is less cell death, as shown by X-gal staining, although migration of progenitor cells to the forelimb bud is compromised. This is illustrated for *Tbx3 *transcripts on *Pax3 *gain (Figure 2C, D) and partial loss (Figure 2E, F) of function backgrounds. *Tbx3 *is also expressed in cardiac neural crest [27], however its expression profile in the somites points to activation in a subdomain of this paraxial mesoderm. Further examples of PCR based analysis of the distribution of Pax3 targets, is shown in Additional file 1 Figure S1. Comparison of *Sox2 *and *Sox10 *transcripts in Pax3 positive cells of different somite preparations indicates that these are high in samples that include the neural tube, consistent with expression in neural crest (Additional file 1 Figure S1B). Comparison of expression in the whole somite (Additional file 1 Figure S1A), with the hypaxial domain is also informative, indicating, for example, that *Zic1 *transcripts are enriched in the whole somite, consistent with an expression mainly in the epaxial domain [28], as seen by immunofluorescence on sections (Additional file 1 Figure S1C), where Zic1 protein is co-expressed with Pax3 in the epaxial dermomyotome as well as in the dorsal neural tube and in Pax3 negative mesenchyme. Pax3 positive neural crest does not appear to express Zic1, in accordance with a recent report on its absence in migratory neural crest cells in the chick embryo [28]. *In situ *hybridization on sections confirms expression of *Zic1 *in the epaxial domain of the epithelial dermomyotome (Additional file 1 Figure S1E). Up-regulation of *Zic1 *transcripts in somites of *Pax3^GFP/GFP ^*embryos in the epaxial/central domain, which is less affected by cell death, is consistent with negative regulation by Pax3 (Additional file 2 Table S2B).

### Cell survival and malignancy

In the absence of Pax3, myogenic progenitor cells undergo apoptosis. This phenotype, in addition to data in adult satellite cells, indicates that Pax3/7 are implicated in cell survival [29]. Our list of Pax3 target genes is not obviously enriched in such genes; however secondary modifications of cell survival proteins are not detected in this approach. Very few genes associated with carcinogenesis emerge as PAX3-FKHR targets. This is in contrast to screens performed in Rhabdomyosarcoma cell lines (for review, see [15]). This may be explained by the physiological level of expression of PAX3-FKHR, similar to that of Pax3 in our screen as well as the *in vivo *context; *Pax3*^*PAX3-FKHR/+ *^mice do not develop tumours unless a second mutation affecting a tumour suppressor occurs [30]. In Rhabdomyosarcoma, a chromosomal translocation has taken place, potentially affecting genome regulation, and the cells examined are derived from an adult tumour, so that the context is different from that of embryonic cells expressing a *Pax3^PAX3-FKHR ^*allele.

In this report we concentrate on signalling pathways and transcription factors implicated in myogenesis.

### Pax3 modulation of signalling pathways

In Table 1 examples of genes encoding components of signalling pathways that are up- (red) or down- (blue) regulated in the presence of PAX3-FKHR in limbs and/or somites are shown. A number of major signalling pathways lie genetically downstream of *Pax3*. In some cases, such as FGF or Eph pathways, transcripts for both ligands and receptors are present in Pax3-GFP positive cells, indicative of autocrine signalling, which is subject to *Pax3 *regulation.

**Table 1:** Changes in transcripts for genes implicated in major signalling pathways between Pax3-GFP/+ samples from somites and/or limbs of gain of function Pax3PAX3-FKHR/GFP and control Pax3GFP/+ embryos: UP (bold), DOWN (italics)

		***Pax3***^***PAX3-FKHR/GFP ***^**vs Pax3**^***GFP/+***^	
**RTK**	FGF	Ligands/Extracellular matrix	**FGF5**, **FGF12**, **Sulfatase1**
		Receptors	**Fgfr4**, *Fgfr3*
		Intracellular components	**Dusp4**, **Fap**
		Inhibitors	**Spry1**, **Spry4**, **Ing5**, **Spred1**
		Transcriptional components	**Etv1**, **Etv2**
	
	IGF	Ligand	**Igf1**
	
	PDGF	Ligand	**Pdgfc**
		Effector (target)	**Csrp1 **(Axud1)
	
	Met	Receptor	**c-Met**
	
	Eph	Ligands	*EphrinA5*, **EphrinB1**
		Receptors	**EphA7**, *EphA3*

**Shh**		Ligands/Extracellular matrix	**Sulfatase1**
		Inhibitors	**Hhip**
		Transcriptional components	*Zic1*

**Wnt**		Ligands/Extracellular matrix	**Wnt16**, **Sulfatase1**
		Intracellular components	**Siah2**, **Diversin **(Ankrd6)
		Inhibitors	*Sfrp3 *(Frzb1), **Dkk1**, **Dkk2**, **Diversin**, **Wif1**, **Csrp1 **(Axud1)
		Transcriptional components	**TCF15**, **TCF7/2 **, *Nkd2*

**Integrins**		Ligands	**Lama2**
		Intracellular components	*Itgβ1bp2*
		Receptors	*Itgβ6*, *Itgβ8*

**Cytokines**		Ligands	**CXC15**, *CXC12*, **Cxcl14**, **Cxcl5**
		Intracellular components	**CXCR7**, **Crlf1**
		Inhibitors	**Soc3**

**Notch**		Ligands	*Dll1*, *Dll3*
		Inhibitors	*Fhl1 *(Kyot2)
		Transcriptional components	**Hes1**

**TGFβ/BMP**		Ligands	**BMP5**, **TGFβ2**, *TGFβinduced*, **Thbs1**
		Inhibitors	**Chordin-like1**, **Follistatin**, **Gremlin**

Transcripts of the enzyme, Sulfatase1, that sulfates extracellular matrix Proteoglycans positively affecting the binding/stability and availability of ligands such as FGF, Wnts and Shh, are up-regulated in the presence of PAX3-FKHR. This is a potentially important level at which signalling, and consequent cell behaviour, is modulated. Indeed in a comparison of quiescent versus *in vivo *activated Pax3 positive muscle satellite cells, expression of genes such as Sulfatase1, affecting extracellular matrix interaction with growth factors, was strikingly modified [31]. In addition to effectors of signalling pathways, inhibitors depend on Pax3 activity. In some cases, due to feedback regulation, activation of a gene for an inhibitor, may reflect activation of a pathway, however it may also demonstrate an important potential for Pax3 modulation of the outcome of signalling, depending on the myogenic context.

Pathways subject to *Pax3 *regulation include receptor tyrosine kinase (RTK) pathways, such as FGF, as well as Shh and Wnt signalling which promote myogenesis. Most effectors of RTK signalling pathways are positively regulated by *Pax3*. Unless specified, regulation is not necessarily direct.

FGF signalling is strikingly affected by Pax3 and *Fgfr4 *has now been shown to be a direct target [19]. Pax3 regulation of *Sprouty1*, encoding an intracellular inhibitor of RTK signalling, has consequences for myogenic progenitor self-renewal, versus entry into the myogenic programme, promoted by this pathway in the embryo [19]. More recently, Sprouty1 has been implicated in controlling quiescence of adult satellite cells [32], although it is not notably modulated *in vivo *in comparisons with activated satellite cells [31]. We investigated *Sprouty1 *mutant embryos [33], in which a *LacZ *reporter permitted clearer identification of *Sprouty1 *expressing cells. In the mutants, expression of the myogenic regulatory genes, *Myf5 *or *MyoD*, viewed by whole mount *in situ *hybridization appears normal (Additional file 1 Figure S2 A-D) and *Desmin*, which marks muscle cells, is expressed in the myotome as expected (data not shown). However *Sprouty2 *and *Sprouty4 *are also expressed in somites [34] (Additional file 1 Figure S2E-F) and may therefore compensate for the absence of Sprouty1. Pax3 regulates multiple components of the FGF signalling cascade, from the ligand/receptor to transcriptional effectors, such as the Ets transcription factors (Etv1, Etv2).

Other RTK pathways, such as IGF and PDGF, also promote myogenesis (for ex [35]); *Igf1 *and *Pdgfc *are up-regulated in the presence of PAX3-FKHR. The *c-Met *gene was one of the first Pax3 targets to be proposed in a myogenic context [7]. In our screen, transcripts for this gene are up-regulated in somites (Additional file 2 Table S2, [9]), but not limb buds (Additional file 2 Table S3), suggesting that Pax3 activation of transcription is confined to the somite (see also Additional file 1 Figure S1), where c-Met is required for delamination [36], although the transcripts (Additional file 3 Table S4) continue to be present in cells that migrate to the limb buds.

A number of genes for Ephrin ligands (EphrinA5, EphrinB1) and receptors (EphA7, EphA3) are regulated by Pax3 in both somites and limbs, suggesting expression in myogenic cells, as well as neural crest [37]. Recently, up-regulation of Eph receptors and ligands has been reported in several Rhabdomyosarcoma cell lines (EphB: [38] EphA [39]). In a myogenic context *in vivo*, Eph receptors have been implicated in muscle patterning and inervation [40,41]. The EphA signalling pathway may also interfere with FGF/MAPK signalling [42,43].

The notochord and ventral neural tube are sources of Shh signalling. In a myogenic context this impacts the adjacent epaxial dermomyotome where *Zic1*, for example, is highly expressed (Additional file 1 Figure S1), and where Shh is implicated in the activation of *Myf5 *[44] as well as in promoting cell survival and proliferation in the somite [45]. Canonical Wnt signalling, from the dorsal neural tube, similarly affects the epaxial somite and *Myf5 *activation [46], with a potential relay through non-canonical Wnts, such as Wnt11 [47]. Components of these pathways are modulated positively or negatively in the presence of PAX3-FKHR suggesting that Pax3 fine-tunes Wnt and Shh signalling, probably also limiting the spatial extent of their action in the somite (see Additional file 1 Figure S1 for Zic1).

Other signalling pathways, such as Notch, that, like FGF, affect self-renewal/differentiation [48,49] show some modulation by Pax3. This is also the case for signalling through Integrins, where the *laminin *gene encoding the ligand, Lama2, is up-regulated (Table 1), also seen for *Lama1 *via *Dmrt2*, which is a Pax3 target [20], whereas transcripts for the Integrin receptors, Itgβ6 and Itgβ8, are down-regulated (Table 1). Integrins, some of which lie genetically downstream of *Myf5*, are important for the structure and myogenic regulation of the dermomyotome and for the formation of the basal lamina that contains the myotome [50].

Transcripts for a number of cytokines and their receptors are present in Pax3 positive cells. Some show modulation by Pax3, although this did not include *CXCR4*, regulated by Lbx1, and important for the migration of a subpopulation of myogenic progenitors into the limb bud [51].

BMP/TGFβ signalling, from the dorsal neural tube and lateral mesoderm, has been shown to antagonise the onset of myogenesis [52]. Again, Pax3 affects this signalling pathway, notably by positively regulating genes encoding both ligands and inhibitors such as Chordin-like1, Follistatin and Gremlin1. In the chick embryo, manipulation of Noggin, produced in the somite, had shown the importance of this inhibitor in permitting *MyoD *activation and the onset of myogenesis [53]. *Gremlin1 *is expressed at the extremities of the dermomyotome at E9.5 (Additional file 2 Table S3A) and subsequently declines in the epaxial domain of more mature somites (Additional file 1 Figure S1B, C) to be no longer detectable by E11.5. It is up-regulated in the presence of PAX3-FKHR (Additional file 3 Figure S3A) (Additional file 1 Figure S3 D, E). In *Gremlin1 *mutant embryos [54], Pax3 expression is normal (results not shown). Somitic expression of *Myf5 *(Additional file 1 Figure S3F, G) and *MyoD *(Figure S3 H, I) is also similar to wild type or heterozygote embryos. However, there is some delay in the onset of expression in the hypaxial domain. There is also a delay in *MyoD *expression in the forelimb bud at E11.5 (Additional file 1 Figure S3), probably reflecting perturbations in signalling pathways within the limb [54]. The lack of a more striking phenotype may reflect compensation by other BMP inhibitors, such as Noggin.

**Table 2:** Changes in transcripts for transcription factors common to somites and forelimbs between Pax3-GFP/+ samples from gain of function Pax3PAX3-FKHR/GFP and control Pax3GFP/+ embryos: UP (bold), DOWN (italics), FC (fold change)

Gene Title	Gene Symbol	FC limb	FC somite
vestigial like 3 (Vito2)	**Vgll3**	8.41	2.22
developing brain homeobox 1	**Dbx1**	6.38	5.19
transcription factor AP-2, gamma	**Tcfap2c**	4.45	1.53
transcription elongation regulator 1-like	**Tcerg1l**	3.61	2.21
Unc4.1 homeobox	**Uncx4.1**	3.36	1.66
PR domain containing 8	**Prdm8**	3.05	1.93
T-box 3	**Tbx3**	2.30	2.16
paired box gene 7	**Pax7**	2.12	1.54
histone deacetylase 5	**Hdac5**	1.97	1.58
myogenic factor 5	**Myf5**	1.90	1.85
nescient helix loop helix 2	*Nhlh2*	-2.06	-0.59
brachyury	*T*	-2.17	-0.55
homeo box A4	*Hoxa4*	-2.64	-1.95
forkhead box C2	*Foxc2*	-3.17	-1.47
homeo box B1	*Hoxb1*	-4.49	-2.33
paired box gene 3	*Pax3*	-4.91	-1.73

**Table 3:** Changes in transcripts for transcription factors from forelimbs only between Pax3-GFP/+ samples from gain of function Pax3PAX3-FKHR/GFP and control Pax3GFP/+ embryos: UP (bold), DOWN (italics), FC (fold change)

Gene Title	Gene Symbol	FC limb
ladybird homeobox 1 homolog corepressor 1	**Lbxcor1**	3.58
nuclear receptor subfamily 3, group C, member 1	**Nr3c1**	2.91
distal-less homeobox 5	**Dlx5**	2.81
myocardin	**Myocd**	2.64
jumonji domain containing 1C	**Jmjd1c**	2.58
runt related transcription factor 1	**Runx1**	2.27
tet oncogene 1	**Tet1**	2.20
H6 homeo box 3 (Nkx5-1)	**Hmx3**	2.00
homeo box A9	*Hoxa9*	-1.82
homeo box A10	*Hoxa10*	-2.01
nuclear receptor subfamily 0, group B, member 1	*Nr0b1*	-2.50
single-minded homolog 2	*Sim2*	-3.16
SRY-box containing gene 2	*Sox2*	-4.04
zinc finger protein of the cerebellum 1	*Zic1*	-4.45
forkhead box G1	*Foxg1*	-11.40

**Table 4:** Changes in transcripts for transcription factors from somites only between Pax3-GFP/+ samples from gain of function Pax3PAX3-FKHR/GFP and control Pax3GFP/+ embryos: UP (bold), DOWN (italics), FC (fold change)

Gene Title	Gene Symbol	FC somites
paired-like homeodomain transcription factor 2	**Pitx2**	3.00
ladybird homeobox homolog 1	**Lbx1**	2.67
trans-acting transcription factor 5	**Sp5**	2.55
zinc finger protein 568	**Zfp568**	2.46
inhibitor of DNA binding 4	**Id4**	2.39
fos-like antigen 2	**Fosl2**	2.34
Kruppel-like factor 4	**Klf4**	2.28
nuclear receptor subfamily 4, group A, member 3	**Nr4a3**	2.26
doublesex and mab-3 related transcription factor like family A2	**Dmrta2**	1.86
inhibitor of DNA binding 2	**Id2**	1.82
ISL1 transcription factor, LIM/homeodomain	**Isl1**	1.75
Kruppel-like factor 11	**Klf11**	1.60
inhibitor of DNA binding 1	**Id1**	1.57
zinc finger, CCHC domain containing 12	**Zcchc12**	1.55
zinc finger, MYND domain containing 11	**Zmynd11**	1.54
LIM homeobox protein 2	**Lhx2**	1.54
transcription factor AP-2 beta	**Tcfap2b**	1.50
basonuclin 2	**Bnc2**	1.45
doublesex and mab-3 related transcription factor 2	**Dmrt2**	1.45
mohawk homeobox	**Mkx**	1.43
histone cluster 2, H3c1	*Hist2h3c1*	-1.42
nuclear receptor co-repressor 2	*Ncor2*	-1.43
forkhead box C1	*Foxc1*	-1.45
runt-related transcription factor 1; translocated to 1	*Runx1t1*	-1.46
homeo box C8	*Hoxc8*	-1.47
zinc finger and BTB domain containing 16	*Zbtb16*	-1.47
similar to COUP-TFI/nuclear receptor subfamily 2, group F, member 1	*Nr2f1*	-1.47
T-box 22	*Tbx22*	-1.47
homeo box C5	*Hoxc5*	-1.51
homeo box C6	*Hoxc6*	-1.51
myocyte enhancer factor 2C	*Mef2c*	-1.52
basic helix-loop-helix family, member e22	*Bhlhe22*	-1.55
achaete-scute complex homolog 1 (Drosophila)	*Ascl1*	-1.56
single-minded homolog 1 (Drosophila)	*Sim1*	-1.58
Meis homeobox 1	*Meis1*	-1.58
dachshund 1 (Drosophila)	*Dach1*	-1.72
chromodomain helicase DNA binding protein 8	*Chd8*	-2.04
myogenic factor 6	*Myf6*	-2.26
myogenin	*Myog*	-2.35
paired-like homeobox 2b	*Phox2b*	-2.39

A striking finding of this screen is the variety of genes for inhibitors of signalling pathways that are controlled by Pax3. These include *Sprouty1*, *Sfrp3*, *Gremlin1 *and *Hhip*, which encode inhibitors of FGF, Wnt, BMP and Shh signalling, respectively (Table 1). This indicates that Pax3 negatively modulates the activity of signalling pathways as well as promoting their activation. The role of the FGF inhibitor, Sprouty, in maintaining the myogenic stem cell population in the face of FGF signalling that promotes entry into the myogenic differentiation programme has been demonstrated [19]. In addition, pathways that negatively impact entry into the myogenic programme, such as BMP/TGFβ, are also abrogated by inhibitors, as illustrated for Gremlin, precisely expressed at the extremities of the dermomyotome where activation of myogenic determination factors is first initiated.

### Pax3 modulation of genes implicated in transcription

Examples of genes involved in the control of transcription that show up- or down-regulation in Pax3 positive (Pax3-GFP) cells in the presence of PAX3-FKHR, compared to controls, are presented in Table 2, 3, 4. This is divided into three sections for differentially regulated genes in both somites (E9.5) and forelimbs (E10.5) (Table 2) or only in forelimbs (Table 3) or only in somites (Table 4). Transcriptional effectors of signalling pathway (see Table 1) have been removed from Table 2, 3, 4.

*Pitx *genes, such as *Pitx2*, which is positively regulated by Pax3, have been implicated in myogenesis [55]. Very few Pitx target genes have been identified to date. Recently, in zebrafish, a member of the *Shroom *family, encoding an actin binding protein implicated in epithelial organization [56,57], has been reported to be a direct target of Pitx factors [58]. Interestingly, *Shroom2*, like *Pitx2*, is up-regulatd by Pax3 in the somite (Additional file 2 Table S2). The *Pax3*-*Pitx2*-*Shroom2 *cascade may be implicated in the maintenance of the epithelial organization of the hypaxial dermomyotome in the mouse embryo.

**Figure 3:**
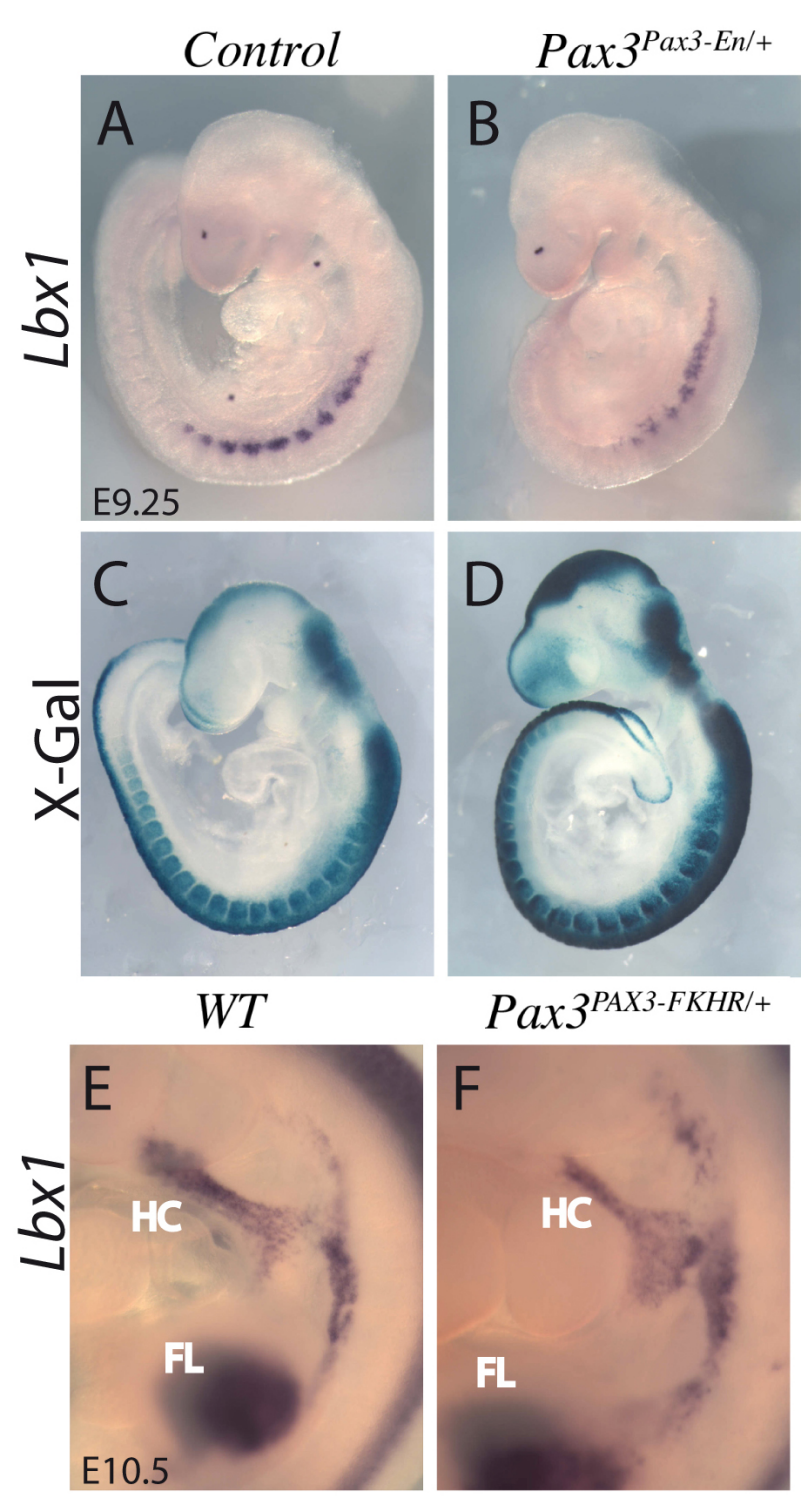
***Lbx1 *expression depends on *Pax3***. (A, B, E, F) Whole mount *in situ *hybridisation with an *Lbx1 *probe of control (A, E), *Pax3^Pax3-En/+ ^*partial loss of function (B) and *Pax3^PAX3-FKHR/+ ^*gain of function embryos (F) at E9.25 (A, B) and E10.5 (E, F), showing down-regulation of *Lbx1 *transcripts when Pax3 activity is reduced, and some up-regulation of its expression in the presence of PAX3-FKHR in somites. In the lower panels (C, D) X-gal staining of β-galactosidase from the *IresnlacZ *reporter in *Pax3^IresnlacZ/+ ^*(C) or *Pax3^Pax3-En-IresnlacZ/+ ^*(D) shows the extent of the somites, notably the hypaxial domain.

*Lbx1 *is another gene that is implicated in myogenesis (Table 4). Previous observations on decreased *Lbx1 *expression in *Pax3 *mutants were difficult to interpret because of cell death in the hypaxial somite, where the gene is expressed at limb levels [1], however the gain of function result shown here indicates that *Pax3 *lies genetically upstream of *Lbx1*. This result was confirmed by whole mount *in situ *hybridization on *Pax3*^*Pax3-En/+ *^partial loss of function embryos at E9.5 (Figure 3B), when apoptosis due to perturbation of Pax3 function is minimal [10] as shown by X-gal staining of Pax3 expressing cells (Figure 3D). *Lbx1 *is also expressed in cells migrating to the limbs, but was not modified in the limb bud microarrays suggesting that, like *c-Met *(which appears as a potential Pax3 target in the dermomyotome but not in the limb), this aspect of its expression is not Pax3 dependent. This result suggests that *Pax3 *regulates the expression of *Lbx1 *and *c-Met *to control cell delamination from the hypaxial dermomyotome of the somite, but not later during progenitor cell migration to the limb buds, thus dissociating its function in delamination and migration. In Pax3 positive cells in the forelimb bud, *Lbxcor1*, which encodes a co-repressor of Lbx [59] is up-regulated (Table 3), suggesting that by this stage repression of Lbx activity is important. In the limbs, the gene for the cytokine receptor, CXCR4, that is required for the development of a subset of myogenic cells, depends on Lbx1 [51]*in vivo*. Results *in vitro *show that expression of *CXCR4 *is regulated by PAX3-FKHR in Rhabdomyosarcoma cell lines [60] and by Pax7 in C2C12 cell lines [13]. However the level of *CXCR4 *transcripts is not altered in the presence of PAX3-FKHR in our screen in the embryo, although they are present in Pax3-GFP positive cells, as expected (Additional file 3 Table S4).

Six homeo-domain transcription factors, with their Eya co-activators and Dach co-repressors, are also important upstream regulators of myogenesis [1]. Transcripts for these factors are present in the Pax3-GFP positive cells (Additional file 3 Table S4), but only *Dach1 *expression is affected by PAX3-FKHR (Table 4); it is down-regulated, in keeping with Pax3 promotion of Six myogenic activity. Manipulation of Dach, which is high in quiescent satellite cells, demonstrates its negative role in activated Pax3-GFP positive cells, retarding their entry into myogenesis [31].

*Sim1 *and *Sim2 *transcripts, that mark hypaxial somite domains and migrating myogenic progenitors [61,62], are both negatively regulated by PAX3-FKHR. Sim2 has been shown to prevent epithelial/mesenchymal transitions (EMT) through repression of *Slug *[63]. When PAX3-FKHR is transfected into NIH3T3 cells, *Slug *transcripts are up-regulated [16]. Pax3 repression of *Sim2 *may be necessary to promote delamination of migratory myogenic cells and indeed in *Pax3^PAX3-FKHR/+ ^*embryos there is premature EMT, accompanied by up-regulation of *c-Met *[9]. Experiments in the chick embryo have shown that FGF signalling from the myotome triggers the expression of Snail, a known regulator of EMT [64]. In our transcriptome data, neither *Snail *nor *Slug *expression was affected and therefore EMT in this context may involve other transcriptional regulators.

Many *Hox *genes (*Hoxa4*, *a9, a10*, *Hoxb1 *and *Hoxc5, c6, c8*), present in somites and/or limbs, are down-regulated in Pax3-GFP positive cells in the presence of PAX3-FKHR. This is an intriguing finding. *Hox *gene regulation at the level of the somites, with consequences for myogenesis, has already been documented [65,66]. Our findings now suggest a reciprocal relationship.

*Foxc2*, is negatively regulated by PAX3-FKHR [21] (Table 2) and this is also the case to a lesser extent for *Foxc1 *in somites (Table 4). Reciprocal negative regulation between *Pax3 *and *Foxc2 *has been implicated in cell fate choices of multipotent cells in the dermomyotome, such that high Pax3 promotes myogenesis at the expense of vasculogenesis and *vice versa *[21]. *Runx1*, and the related gene *Runx1t1*, are up-regulated in the presence of PAX3-FKHR in forelimb buds and somites respectively (Table 2, 3, 4). Runx1 is a factor that marks endothelial cells, some of which, in the limb, derive from the dermomyotome [2]. This would suggest that Pax3 may contribute to the priming of cells to become endothelial, although it is Foxc2 that promotes the vascular fate. *Myocardin *is also up-regulated, indicating positive control by Pax3 (directly or indirectly). Myocardin controls smooth muscle differentiation [67] and this may also be indicative of such "priming". However some smooth muscle markers are also expressed in differentiating skeletal muscle cells in the embryo. Unexpected expression of *Myocardin *in the dorsal somite had already been reported [67].

The gene that encodes the myogenic determination factor, Myf5, is up-regulated by PAX3-FKHR, both in the somite at E9.5 and in E10.5 forelimb buds (Table 2). This is consistent with direct activation by Pax3 of the *Myf5 *limb regulatory element [10] and of regulation of early *Myf5 *expression through *Dmrt2*, which is itself a Pax3 target in the dermomyotome in the epaxial and potentially also the hypaxial domain [20] (Table 4). Interestingly the gene for the transcriptional co-factor Vgll3 (also called Vito-2) is up-regulated (Table 2 see also Figure 2B). Vgll3, expressed at the onset of myogenesis [68], enhances the transcriptional activity of TEF transcription factors that bind to MCAT motifs, present in many skeletal muscle specific genes [69]. In this context, Six homeodomain proteins, in addition to their upstream role in concertation with Pax3 at the onset of myogenesis, also, unlike Pax3, directly activate differentiation genes. Down-regulation of the gene for the Six co-repressor, Dach1 will also promote differentiation. However genes encoding transcription factors associated with myogenic differentiation, such as *Myogenin*, *Mrf4*, *Mef2c *and downstream muscle genes, such as *Myosins *or *Troponins *are down-regulated in the presence of PAX3-FKHR (Additional file 2 Table S3B). Activation of myogenic differentiation may be prevented by negative regulation in Pax3 expressing cells of *Meis1 *(Table 4), which encodes a protein, required for chromatin accessibility in a myogenic context, as shown for MyoD [70]. *Mbnl3 *(Muscleblind-like 3), up-regulated by PAX3-FKHR (Additional file 2 Table S3A, Figure 2B), encodes a protein that inhibits MyoD dependent gene expression, thus antagonising differentiation [71]. In this context, Myocardin in concertation with Hdac5, also modulated by Pax3 (see below), represses MyoD/Myf5 activation of the *Myogenin *promoter [72], thus preventing skeletal muscle differentiation. Myocardin expression, also detected in the dermomyotome [72], may prevent premature differentiation of Myf5 expressing cells in the hypaxial domain. Furthermore differentiation will be reduced by the positive effect of Pax3 on the expression of *Id1*, *Id2 *and *Id4 *(Table 4), encoding helix-loop-helix proteins which complex with basic helix-loop-helix factors such as MyoD, interfering with DNA binding [73]. *Id3 *did not emerge from our screen, but this gene had been identified as a Pax3 target in cultured muscle cells [14]. Overexpression of Pax7 in cultured muscle cells identified *Id2*, as well as *Id3*, as a target [13]. In the embryo, targeting of *Pax3 *alleles with a Pax7 coding sequence [12] showed that Pax7 can replace Pax3 and therefore that these genes share common targets. These observations indicate that while Pax3 is required for entry into the myogenic programme, it also acts as a brake on muscle differentiation and indeed continued high level of expression of Pax3 retards the onset of differentiation in muscle satellite cells [74].

**Figure 4:**
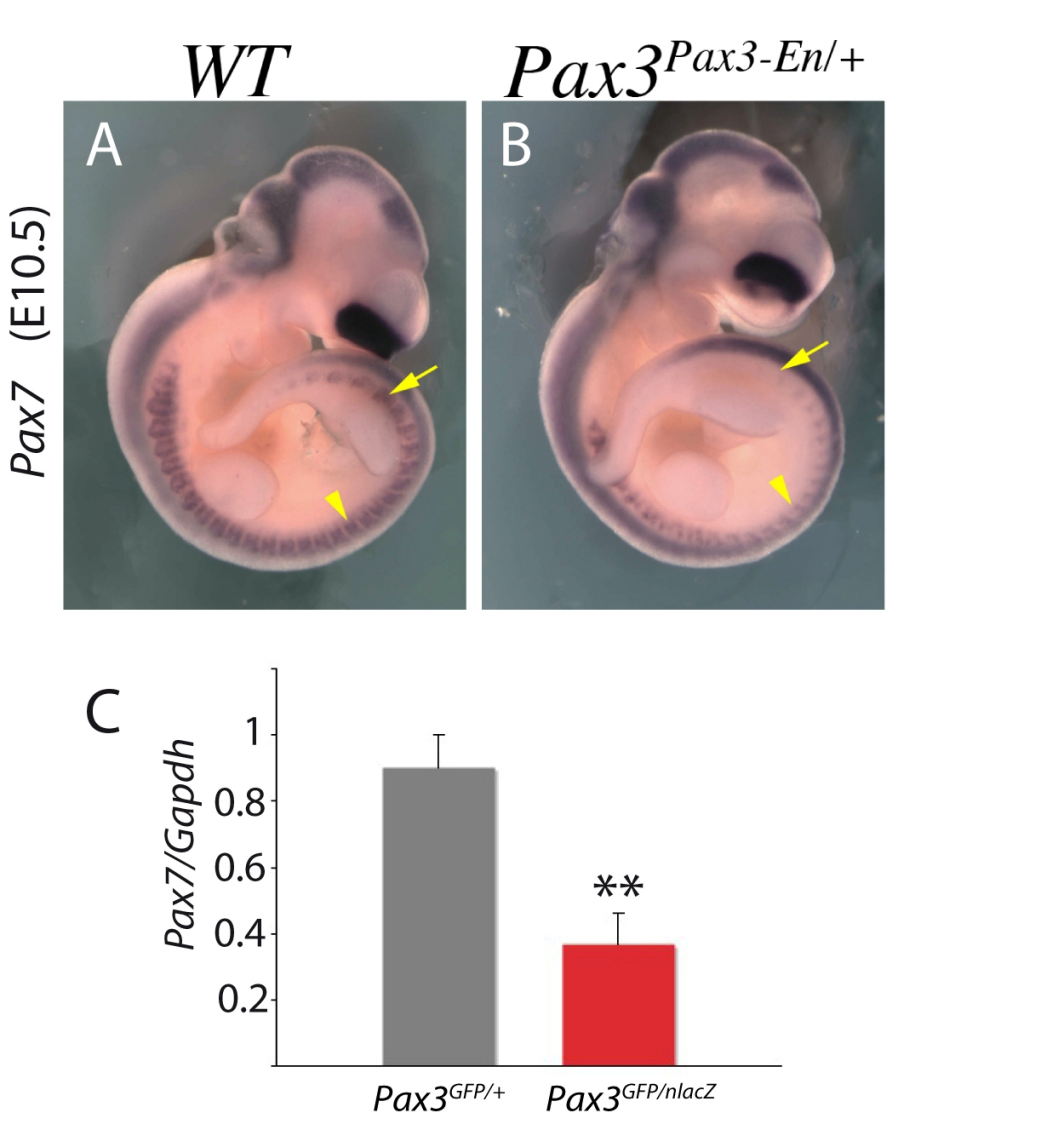
***Pax7 *expression depends on *Pax3***. (A, B) Whole mount *in situ *hybridisation with a *Pax7 *probe of wild-type (WT) (A) and *Pax3^Pax3-En/+ ^*partial loss of function (B) embryos at E10.5, showing down-regulation of *Pax7 *transcripts when Pax3 activity is reduced. (C) GFP positive cells were isolated by flow cytometry (see Figure 2) from the interlimb somites of *Pax3^GFP/+ ^*and *Pax3^GFP/nlacZ ^*embryos at E10.5. The same number of cells in each case was used for RNA preparation and quantitative PCR analysis, with *Pax7 *and *Gapdh *primers; mRNA levels are shown relative to *Gapdh *transcripts with the heterozygote control level taken as 1. Histograms represent mean fold change for four different biological samples analyzed in duplicate at each stage; error bars, standard error to the mean (SEM); **p < 0.005.

Transcripts of *Pax7*, the paralogue of *Pax3*, are higher in the presence of PAX3-FKHR in both limbs and somites (Table 2). Down-regulation of *Pax7 *in somites, when Pax3 activity is reduced, is seen by whole mount *in situ *hybridization with a *Pax7 *probe on *Pax3^Pax3-En/+ ^*embryos (Figure 4B). When quantitative PCR is performed on RNA isolated from the same numbers of Pax3-GFP cells purified by flow cytometry from *Pax3^GFP/+ ^*and *Pax3^GFP/nlacZ ^*embryos, the reduction of *Pax7 *transcripts in the absence of Pax3 is striking (Figure 4C, D). This observation on Pax3 dependence of *Pax7 *expression is important in understanding Pax3/7 regulation of muscle stem cell fate. This *in vivo *result is in contrast to *in vitro *observations where PAX3-FKHR overexpression in human Rhabdomyosarcoma cell lines leads to a down-regulation of *PAX7 *expression [60]. It has also been reported that *Pax7 *is up-regulated in the somites of *Pax3 *mutant (*Splotch*) embryos [75], however this was not a quantitative analysis, and somite disorganisation in the absence of Pax3 complicates the interpretation of *in situ *hybridisation.

**Figure 5:**
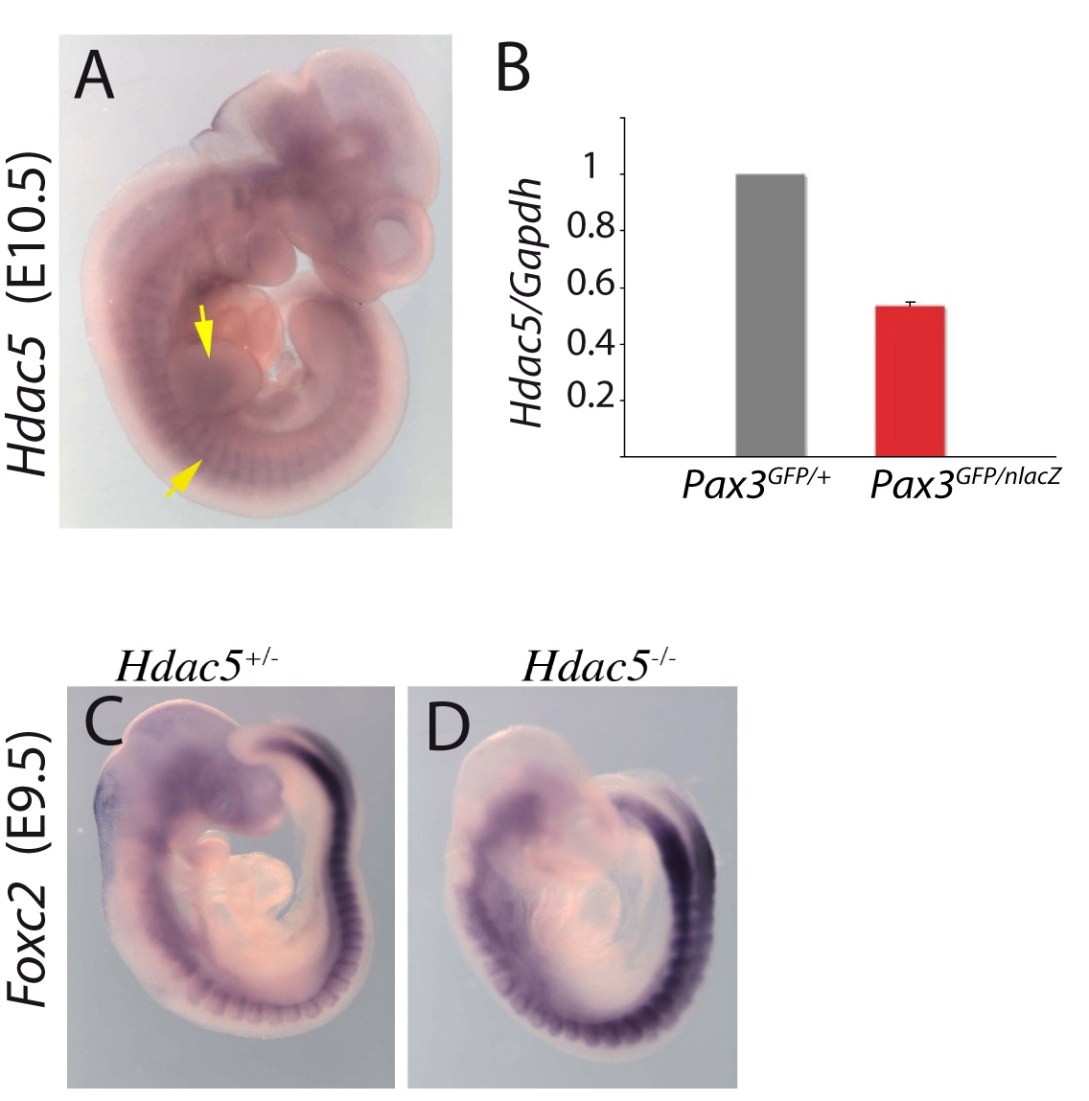
**The *Hdac5 *gene is positively regulated by *Pax3 *and negatively affects *Foxc2***. (A) Whole mount *in situ *hybridisation at E10.5 with an *Hdac5 *probe. Arrows point to expression at sites of myogenesis in the somites and forelimb bud. (B) GFP positive cells were isolated by flow cytometry (see Figure 2) from interlimb somites of *Pax3^GFP/+ ^*and *Pax3^GFP/nlacZ ^*embryos and the same numbers of cells were used for RNA preparation and quantitative PCR analysis of *Hdac5 *and *Gapdh *transcripts; mRNA levels are shown relative to *Gapdh *transcripts with the heterozygote control (*Pax3^GFP/+^*) level taken as 1. Histograms represent mean fold change for two different biological samples analyzed in duplicate at each stage; error bars, standard error to the mean (SEM). (C-D) Whole mount *in situ *hybridisation with a *Foxc2 *probe on *Hdac5^+/- ^*(C) and *Hdac5^-/- ^*(D) embryos at E9.5, showing up-regulation of *Foxc2 *in somites in the absence of Hdac5.

Finally, the gene encoding the histone deacetylase, Hdac5, a class II histone deacetylase, that acts as a negative regulator of transcription, is up-regulated in Pax3-GFP positive cells in the presence of PAX3-FKHR in somites and limb buds (Table 2). Expression of *Hdac5 *at these sites of myogenesis is shown by whole mount *in situ *hybridization in Figure 5A and confirmation that it is positively regulated by Pax3 is shown by qRT-PCR on RNA from the same number of Pax3-GFP positive cells (Figure 5B). Pax3 acts mainly as a transcriptional activator in the myogenic context [9] and therefore genes that are down-regulated in a *Pax3 *gain of function context are probably indirectly regulated by Pax3. *Foxc2 *is an example of such a gene [21]. Hdac5 is a candidate negative intermediary. In *Hdac5 *mutants, *Foxc2 *is up-regulated (Figure 5D), indicating that *Pax3 *repression of *Foxc2 *may be mediated by *Hdac5*, which is itself positively regulated by Pax3.

## Conclusions

We have identified sequences that are potential Pax3 targets, thus giving insight into Pax3 orchestration of progenitor cell behaviour prior to, and at the onset of, myogenesis. Many components of signalling pathways, including inhibitors as well as activators, emerge from the screen, demonstrating how Pax3 modulates their impact on progenitor cell behaviour and progression towards muscle. This is also evident from transcriptome analysis of chromatin remodelling and transcription factors/co-factors. *Pax3 *regulated sequences modulate initial cell fate decisions in the multipotent Pax3 positive stem cells of the dermomyotome. In this case *Hdac5*, positively regulated by *Pax3*, negatively impacts *Foxc2 *expression. *Foxc1 *is also down-regulated indirectly by *Pax3*. In this stem cell context, *Pax3 *positively regulates *Pax7 *also implicated in reciprocal repression with *Foxc1/c2 *[21]. Entry into the myogenic programme is promoted by down-regulation by *Pax3 *of the gene for the Six1/4 co-repressor Dach2 and also by the previously demonstrated activation of the myogenic determination gene *Myf5*, which in this case has been shown to be direct [10]. Genes for myogenic differentiation factors and downstream muscle proteins are mainly down-regulated by *Pax3*, acting negatively on the gene for the chromatin remodelling factor Meis and positively on the *Id *gene family of myogenic inhibitors as well as on *Myocardin*. Pax3, either directly or indirectly, is thus acting as a brake on muscle differentiation, while priming entry into the myogenic programme. Regulation of myogenic progenitor cell behaviour, both at the level of signalling pathways and of transcriptional control, is modulated by balanced up- and down-regulation of genes that lie genetically downstream of *Pax3*.

## Methods

### Mouse lines

The following mouse lines were used: *Pax3^GFP/+^*, *Pax3^PAX3-FKHR-IRESnlaZ/+ ^*(referred to as *Pax3^PAX3-FKHR/+^*), *Pax3^Pax3-En-IRESnlacZ/+ ^*(referred to as *Pax3^Pax3-En/+^*), *Pax3^nLacZ/+^, Hdac5^nlacZ/+^*, *Sprouty1^lacZ/+^*, *Gremlin1^+/- ^*and the *PGK-Cre *transgenic line. Embryos were genotyped as described previously: *Pax3^GFP/+ ^*[6,10], *Pax3*^*PAX3-FKHR *^and *Pax3*^*nLacZ *^[9], *Pax3^Pax3-En ^*[10], *PGK-Cre *[22], *Hdac5^nlacZ/+ ^*[76], *Sprouty1^lacZ/+ ^*[33], *Gremlin1^+/- ^*[54]. The targeted *Pax3 *lines used in this analysis have been bred for many generations on a C57 BL6/DBA2 genetic background.

For the screen, *Pax3^GFP/+ ^*mice were crossed with *PGK-Cre *transgenic mice to obtain *Pax3^GFP/+^*; *PGK-Cre *females. These females were crossed with *Pax3^PAX3-FKHR-IRESnlacZ/+ ^*males to obtain embryos with one *Pax3^GFP ^*allele and one floxed *Pax3^PAX3-FKHR-IRESnlacZ ^*allele [9].

### Preparation of embryonic material for *in situ *gene expression and micro-array analysis

Embryos were collected after natural overnight mating and dated, taking Embryonic day (E) 0.5 as the day after the appearance of the vaginal plug. Briefly, embryos were fixed in 4% para-formaldehyde at 4°C, overnight for *in situ *hybridization, 2 hours for immuno-detection and 15 minutes for X-Gal staining.

Embryos were dissected in DMEM medium. For tissue preparation from E9.5 embryos, somites were dissected from the interlimb region and the more hypaxial domain separated from the neural tube and epaxial extremity of the somites. An effort was made to take the epithelial dermomyotome, viewed by Pax3-GFP fluorescence under the microscope. The forelimb buds at E10.5 were separated from the adjacent somites under a fluorescence microscope. Only 1000 cells were collected per limb bud from a *Pax3*^*GFP/+ *^embryo, so that a total of 490 embryos were dissected, 125 of which were *Pax3^PAX3-FKHR/GFP ^*and 107 were *Pax3^GFP/+^*. The genotype was revealed by GFP fluorescence and characteristic head and neural tube abnormalities in *Pax3^PAX3-FKHR/GFP ^*embryos [9], as well as by β-galactosidase activity shown by X-gal staining of the rest of the embryo (from the *Pax3*^*PAX3-FKHR *^allele). The limb buds and hypaxial somites were pooled according to their genotype and then dissociated by passage through a 2 ml syringe and filtered before the flow cytometry sorting.

Triplicate samples of each population were prepared, representing a starting material of a minimum of 100,000 cells per sample. GFP+ cells were separated by flow cytometry using a MoFlo cell sorter (Beckman-Coulter USA). The gates for positive and negative GFP cells were determined using an equivalent sample isolated from wild type embryos. Analysis was done with the Summit software version 3.4.

### RNA isolation and microarray analysis

Total RNA was extracted and purified after DNase 1 (Amersham) treatment using the RNeasy Mikro kit (Qiagen). RNA and cRNA quality was monitored on Agilent RNA Pico LabChips (Agilent). cRNA obtained from 100 ng of RNA was amplified by using the GeneChip Expression Two-Cycle 3'amplification system (Affymetrix). Fragmented biotin-labeled cRNA samples were hybridized on GeneChip Mouse Genome 430_2 arrays, according to the manufacturer's protocol (http://www.affymetrix.com/support/downloads/manuals/expression analysis technical manual.pdf). The Affymetrix 430.2.0 mouse array that contains 45,000 probe sets was used. Each probe set consists of 22 probes of 25 bp, with 11 perfect matches and 11 mismatches. For each experimental group (*Pax3^+/+^*, *Pax3^GFP/+ ^*and *Pax3^PAX3-FKHR/GFP^*), three biological replicates were hybridized. The generation of cell intensity files and the quality control of hybridizations were performed with GeneChip Operating Software (Affymetrix).

### Statistical analysis of microarray data

Statistical analyses of data were performed as described previously [31].

Raw data were pre-processed using the Robust Multichip Analysis (RMA) algorithm in order to correct the background, to adjust the intensity distribution over the arrays and to convert probe intensity summarisation into a unique probe set signal. Unreliable probe-sets called "absent" by Affymetrix GCOS software for at least 2 GeneChip out of 3 were discarded. Local Pooled Error (LPE) tests [23] were performed in order to identify significant differences in gene expression between Pax3-GFP positive cells from *Pax3^PAX3-FKHR/GFP ^*and *Pax3^GFP/+ ^*embryos on the one hand and between GFP+ and GFP- samples from *Pax3^GFP/+ ^*embryos on the other hand. The Benjamini-Hochberg (BH) multiple correction test [24] was applied to control for the number of false positive with an adjusted 5% statistical significance threshold. The fold changes of the differentially expressed genes after p-value adjustement were analyzed by filtering the data set with a threshold of 1.5 (Log2 ratio = 0.5). Significantly regulated genes in *Pax3^PAX3-FKHR/GFP ^*samples, that are common to both limb and somite extracts, are represented in Additional file 2 Table S1. Transcripts that are specifically up- or down-regulated in the limb or in the trunk, respectively, are referred to as "somite only" in Additional file 2 Table S2 and "limb only" in Additional file 2 Table S3.

Genes that are specifically transcribed in the GFP positive fraction (absent in GFP negative fraction) are represented in Additional file 3 Table S4, again as common to somites and limbs (A), somite specific sequences (B) and limb specific sequences (C).

### Accession Numbers

The complete microarray data have been deposited in NCBI's Gene Expression Omnibus and are accessible through GEO series accession number GSE22041 (http://www.ncbi.nlm.nih.gov/geo/query/acc.cgi?acc=GSE22041).

### In situ hybridization

Whole-mount *in situ *hybridizations with digoxigenin-labeled probes were performed as described in [77]. *In situ *hybridization for *Pax7 *transcripts was carried out as described in [12] and for transcripts of *Foxc2 *as described in [21]. The *Tbx3 *probe was as described in [27]. The *Zic1 *probe was synthesized using the image clone Image 4314316 (Open Biosystems) and linearised by EcoR1 and transcribed using T3 polymerase. The mouse *Grem1 *cDNA (containing the complete coding region and 3-UTR) was isolated by RT-PCR from cDNA of RNA prepared from C57BL/6 mouse embryos at E9.5. The *Grem1 *cDNA was subcloned into pBS digested with EcoRl and BamHl and transcribed using T3 polymerase for *in situ *hybridization.

When needed, the whole-mount stained embryos were embedded into gelatin-sucrose, frozen and sectioned, as described in [19].

### Quantitative and semi-quantitative real-time PCR

RNA was extracted from embryonic material (interlimb somites) and reverse transcribed using SuperScript II kit (Invitrogen) for qRT-PCR and SuperscriptIII kit for semi-quantitative RT-PCR. All PCR reactions were carried out in duplicate (triplicate for the standard curves) using the Power Sybergreen Mix (Applied Biosystems) and a 7500 thermal cycler (Applied Biosystems). All qPCR results are expressed as relative ratios of the target cDNA to *Gapdh *transcripts normalized to that ratio in the reference condition, which always corresponds to heterozygote *Pax3*^*GFP/+ *^embryos. Primers used for detecting specific transcripts were designed with Primer3 (see Additional file 4 Table S5).

### Immunofluorescence

Fluorescent co-immunohistochemistry on sections was carried out as described previously [19]. The following antibodies were used: anti-Zic1 (Abcam, ab7524-25), 1/500; anti-Pax3 (DSHB), 1/250. Images were acquired with Apotome Zeiss and Axiovision 4.6 software at the Pasteur imaging center (Imagopole, Institut Pasteur).

Mouse work was carried out in accordance with the regulations of the French Ministry of Agriculture, as practised by the Ministry accredited mouse animal house of the Pasteur Institute under the supervision of scientists and technicians with the official authorisation to experiment on mice. The authors have paid attention to the ARRIVE and MIQE guidelines, in reporting their work.

## Authors' contributions

ML conducted experiments, discussed results and wrote a first draft. TS conducted and discussed experiments. BR performed the statistical analyses of the data. AC helped with the flow cytometry sorting. JL and AZ provided transgenic embryos. FR made the *Pax3 *alleles and initiated the study when in MB's laboratory and continued to supervise the work of ML thereafter. MB directed the work, discussed results and wrote the manuscript.

The manuscript has been approved by all the authors.

## Supplementary Material

Additional file 1 **Supplementary Figures S1-S3**. Figure S1: Examples of transcript validation and analysis of the expression of *Zic1*, negatively regulated by *Pax3*. (A, B) Examples of the analysis of transcripts in different Pax3-GFP cell populations isolated from *Pax3^GFP/+ ^*embryos at E10.5. (A) RT-PCR analysis of RNA extracted from FACs sorted GFP positive and GFP negative cells from *Pax3*^*GFP/+ *^embryos, as indicated in Figure 1 showing that transcripts for the neural tube markers, *Sox2 *and *Sox10*, which are potential Pax3 targets in neural crest, since the transcripts are enriched in whole somite preparations which include neural tissue. (B) RT-PCR analysis of transcripts in GFP positive cells from the hypaxial versus whole somites of *Pax3*^*GFP/+ *^embryos. *c-Met*, *Six1*, *Sim1 *and *Zic1 *transcripts are shown, with *HPRT *transcripts as a control. *Zic1 *expression is higher in the whole somite preparation, consistent with a more epaxial location. (C) Immunohistochemistry with Zic1 and Pax3 antibodies on a transverse section of an interlimb somite of an E10.5 embryo, showing Zic1 protein mainly detected in Pax3 positive epithelial cells of the epaxial dermomyotome (Ep), as well as in the dorsal neural tube (NT) and in mesenchymal cells between the neural tube and somites. Zic1 is mainly absent from Pax3 positive migratory neural crest in this region. (D, E) Whole mount *in situ *hybridization with a *Zic1 *probe on posterior somites of control *Pax3^GFP/+ ^*(D) and mutant *Pax3^GFP/GFP ^*(E) embryos at E11, showing up-regulation in the absence of Pax3. Cell death is extensive in the hypaxial domain of more anterior somites by this stage, but despite some loss of cells in immature posterior somites, *Zic1 *hybridisation is still higher in the *Pax3 *mutant. (F, G) Transverse sections of immature posterior somites from *Pax3^GFP/+ ^*(F) and *Pax3^GFP/GFP ^*(G) embryos at E11. In this tail region, immature somites have not yet undergone cell death. In the control (F), transcripts are concentrated in the more central and epaxial domain whereas in the *Pax3 *mutant (G) they are detected throughout the somite. NT, neural tube. Figure S2: The onset of myogenesis in *Sprouty1 *mutants and reduction of *Sprouty2 *expression in the absence of Pax3. (A-D) Whole mount *in situ *hybridisation on *Sprouty1^lacZ/+ ^*(A, C) and *Sprouty1^lacZ/- ^*(B, D) embryos at E10 (somite stages (ss) are indicated), using *Myf5 *(A, B) and *MyoD *(C, D) probes. FL, forelimb bud. (E, F) *In situ *hybridisation with a *Sprouty2 *probe on E9.5 (E), and with a *Sprouty4 *probe on E10.5 (F), embryos showing interlimb somites and limb buds. There is extensive *Sprouty *expression in the distal limb buds, but positive Sprouty4 positive myogenic progenitors in the proximal forelimb bud are detectable at E10.5 (arrow). Figure S3: Expression of *Gremlin1 *and perturbation of *MyoD *activation in *Gremlin1 *mutant embryos. (A, B, D-I) Whole mount *in situ *hybridisation on wild type (A, B), control (*PGK-Cre*) (D, D'), *Pax3^PAX3-FKHR/+ ^*(E, E'), *Gremlin1^+/- ^*(*Grem1^+/-^*) (F, H) and *Gremlin1^-/- ^*(G, I) embryos at the stages indicated (ss, somite stage), using *Gremlin1 *(Grem1) (A-E'), *Myf5 *(F, G) and *MyoD *(H, I) probes. (C) Transverse section of the embryo shown in (B) at the level indicated by the white bar in (B). Note the expression of *Grem1 *in the hypaxial domain of the dermomyotome (Hyp). NT, neural tube; DA, dorsal aorta; FL, forelimb bud; HL, hindlimb bud. Arrows and arrowheads point to differences in expression in forelimb buds and more posterior somites, respectively. Click here for file

Additional file 2 **Tables S1-S3**. In all tables, the probe name corresponds to the probe set designed by Affymetrix. In some cases, multiple probe sets correspond to the same transcript, the name of which is indicated in the gene title and gene symbol columns. When a probe set has not yet been annotated, a - sign has been assigned or the corresponding Riken number. The Fold change (FC) corresponds to the difference in signal intensities between *Pax3^PAX3-FKHR/GFP ^*and *Pax3^GFP/+ ^*embryos. This number is not on a logarithmic scale. The Adjusted (Adj) p-Value is also indicated. Table S1: Transcripts up-regulated (Table 1S-A) or down-regulated (Table 1S-B) in *Pax3^PAX3-FKHR/GFP ^*(in comparison with *Pax3^GFP/+ ^*embryos) in both forelimb and somite extracts. Table S2: Transcripts up-regulated (Table S2-A) or down-regulated (Table S2-B) in *Pax3^PAX3-FKHR/GFP ^*(in comparison with *Pax3^GFP/+ ^*samples) in forelimb buds. Table S3: Transcripts up-regulated (Table S3-A) or down-regulated (Table S3-B) in *Pax3^PAX3-FKHR/GFP ^*(in comparison with *Pax3^GFP/+ ^*samples) in somites. Click here for file

Additional file 3 **Table S4**. Transcripts that are specifically expressed in GFP positive muscle progenitor cells and that are not detected in the GFP negative population of the forelimb bud. This list of transcripts corresponds to myogenic progenitor cell markers. In this table, mean intensity, not fold change ((shown in Tables S1-3), is represented. Click here for file

Additional file 4 **Table S5**. Sequence of reverse (Rev) and forward (Fwd) primers used for semi-quantitative and quantitative PCR. Click here for file
