# Supplementary figures and images for: Transcriptome analyses based on genetic screens for Pax3 myogenic targets in the mouse embryo
Source: BMC Genomics. 2010 Dec 8;11:696. doi: 10.1186/1471-2164-11-696 (PMC3018477; doi:10.1186/1471-2164-11-696)

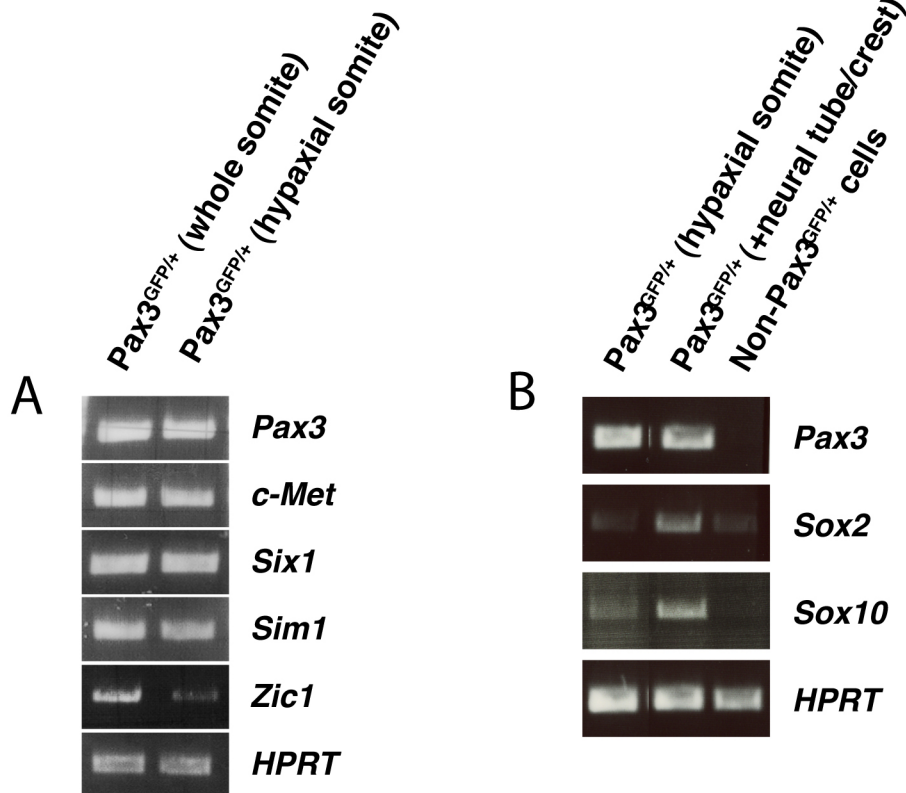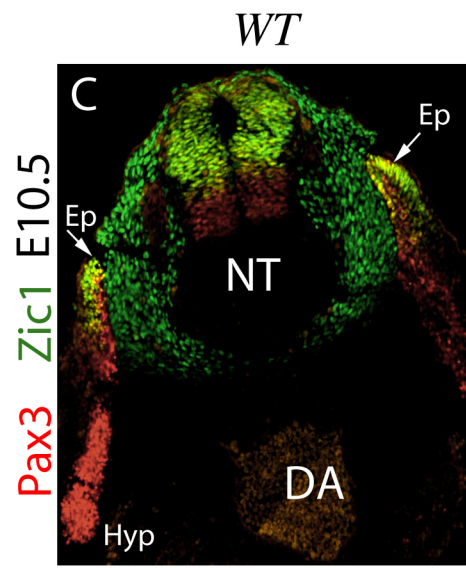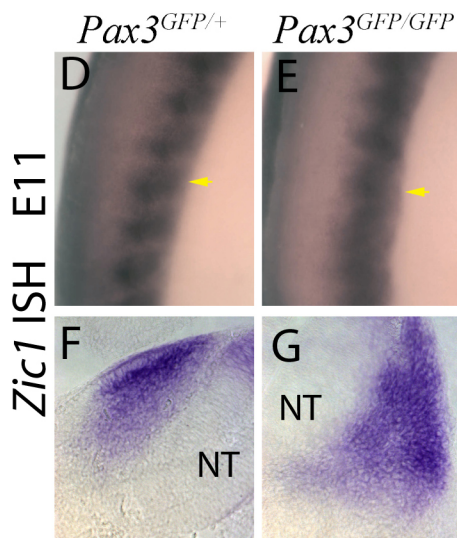

Figure S2

*Sprouty1*<sup>lacZ/+</sup>

*Sprouty1*<sup>lacZ/-</sup>

*Myf5*

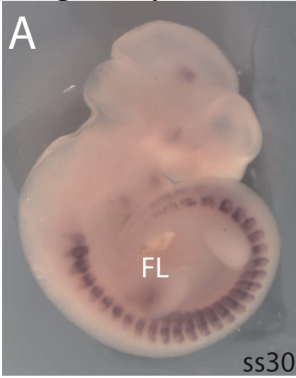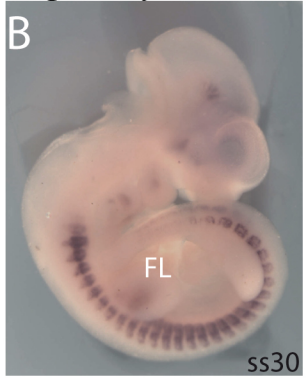

*MyoD*

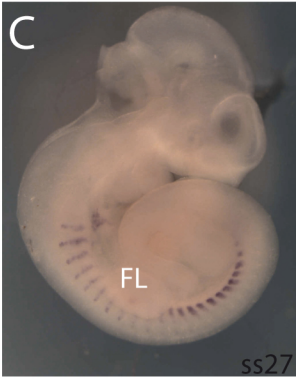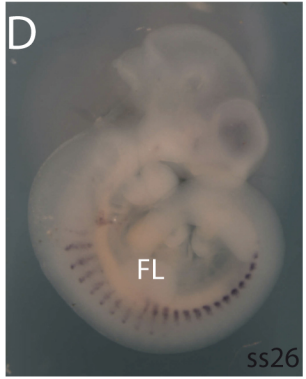

WT

WT

*Sprouty2*

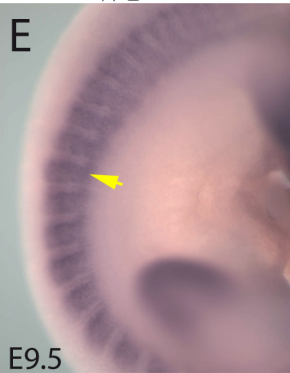

*Sprouty4*

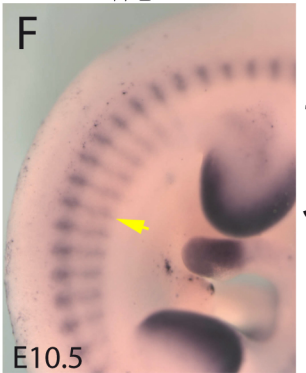

**FigureS3**

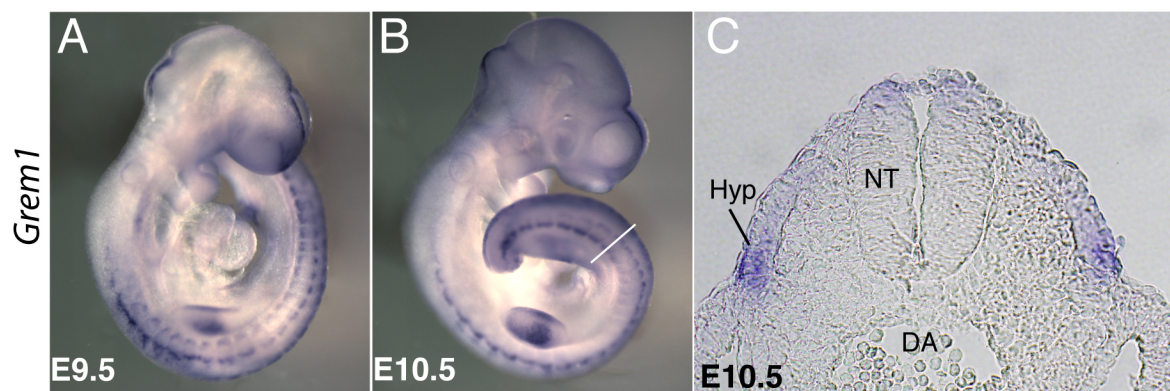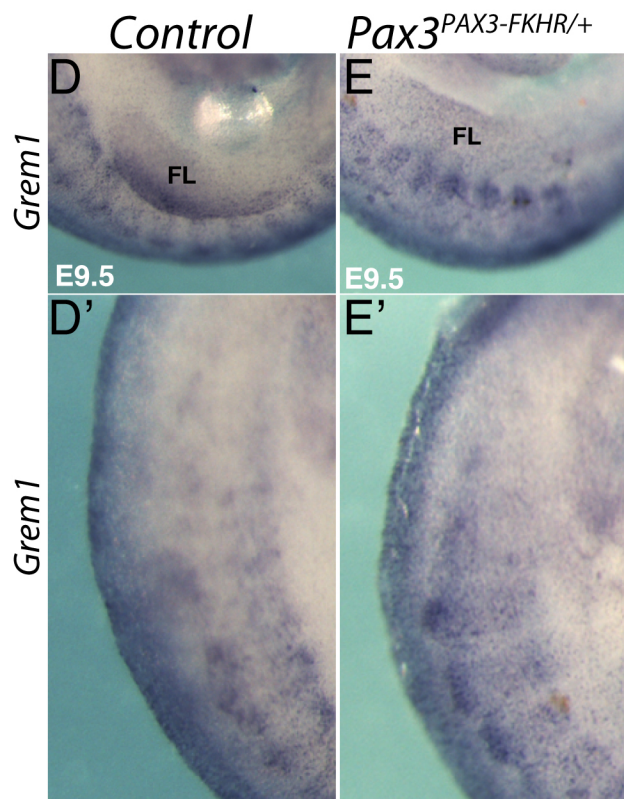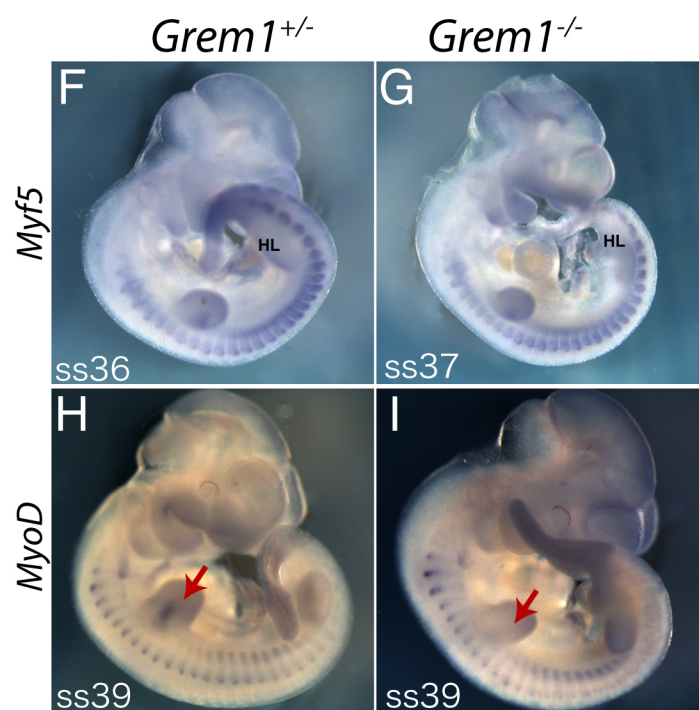

Supplement: Additional file 1 — Supplementary Figures S1-S3. Figure S1: Examples of transcript validation and analysis of the expression of Zic1, negatively regulated by Pax3. (A, B) Examples of the analysis of transcripts in different Pax3-GFP cell populations isolated from Pax3GFP/+ embryos at E10.5. (A) RT-PCR analysis of RNA extracted from FACs sorted GFP positive and GFP negative cells from Pax3GFP/+ embryos, as indicated in Figure 1 showing that transcripts for the neural tube markers, Sox2 and Sox10, which are potential Pax3 targets in neural crest, since the transcripts are enriched in whole somite preparations which include neural tissue. (B) RT-PCR analysis of transcripts in GFP positive cells from the hypaxial versus whole somites of Pax3GFP/+ embryos. c-Met, Six1, Sim1 and Zic1 transcripts are shown, with HPRT transcripts as a control. Zic1 expression is higher in the whole somite preparation, consistent with a more epaxial location. (C) Immunohistochemistry with Zic1 and Pax3 antibodies on a transverse section of an interlimb somite of an E10.5 embryo, showing Zic1 protein mainly detected in Pax3 positive epithelial cells of the epaxial dermomyotome (Ep), as well as in the dorsal neural tube (NT) and in mesenchymal cells between the neural tube and somites. Zic1 is mainly absent from Pax3 positive migratory neural crest in this region. (D, E) Whole mount in situ hybridization with a Zic1 probe on posterior somites of control Pax3GFP/+ (D) and mutant Pax3GFP/GFP (E) embryos at E11, showing up-regulation in the absence of Pax3. Cell death is extensive in the hypaxial domain of more anterior somites by this stage, but despite some loss of cells in immature posterior somites, Zic1 hybridisation is still higher in the Pax3 mutant. (F, G) Transverse sections of immature posterior somites from Pax3GFP/+ (F) and Pax3GFP/GFP (G) embryos at E11. In this tail region, immature somites have not yet undergone cell death. In the control (F), transcripts are concentrated in the more central and epaxi [file 1471-2164-11-696-S1.PDF]
